# RNA-Binding Protein ZFP36L2 Downregulates Helios Expression and Suppresses the Function of Regulatory T Cells

**DOI:** 10.3389/fimmu.2020.01291

**Published:** 2020-06-23

**Authors:** Sohei Makita, Hiroaki Takatori, Arifumi Iwata, Shigeru Tanaka, Shunsuke Furuta, Kei Ikeda, Akira Suto, Kotaro Suzuki, Silvia B. V. Ramos, Hiroshi Nakajima

**Affiliations:** ^1^Department of Allergy and Clinical Immunology, Graduate School of Medicine, Chiba University, Chiba, Japan; ^2^Department of Rheumatology, Hamamatsu Medical Center, Shizuoka, Japan; ^3^Department of Biochemistry and Biophysics, University of North Carolina at Chapel Hill, Chapel Hill, NC, United States

**Keywords:** ZFP36L2, Helios, regulatory T cells, AU-rich element, 3′untranslated region

## Abstract

The zinc finger protein 36-like 2, ZFP36L2, is a member of a small family of RNA-binding proteins composed by ZFP36 (also known as tristetraprolin, TTP), ZFP36L1 and ZFP36L2 in humans, with corresponding murine orthologs. These proteins bind to adenine uridine-rich element (ARE) in the 3′untranslated region of target messenger RNA and stimulate target degradation. ZFP36 functions as an anti-inflammatory modulator in murine models of inflammatory diseases by down-regulating the production of inflammatory cytokines such as tumor necrosis factor-α. However, how ZFP36L1 and ZFP36L2 alter the function of CD4^+^ T cells is not completely understood. We addressed this issue by searching for the target genes of ZFP36L2 by comprehensive transcriptome analysis. We observed that ZFP36L2 is highly expressed in naïve CD4^+^ T cells; however, when CD4^+^ T cells are stimulated through their T cell receptors, ZFP36L2 expression is rapidly reduced in both humans and mice. Among CD4^+^ T cell populations, the expression levels of ZFP36L2 in regulatory T cells (Tregs) were significantly lower than those in naïve or effector CD4^+^ T cells. RNA-sequence analysis revealed that the forced expression of ZFP36L2 decreased *Ikzf2* (encoding Helios) expression in Foxp3^+^ Tregs and inhibited the ability of induced Tregs (iTregs). ZFP36L2 directly bound to and destabilized the 3′untranslated region of *Ikzf2* mRNA, which contains AU-rich elements. These results indicate that ZFP36L2 reduces the expression of *Ikzf2* and suppresses iTreg function, raising the interesting possibility that the inhibition of ZFP36L2 in iTregs could be a therapeutic strategy for autoimmune diseases.

## Introduction

Zinc finger protein 36 (ZFP36) family proteins contain a similar tandem zinc finger domain, composed of three cysteines and one histidine. Because their tandem zinc finger domain can bind to adenine-uridine rich elements (AREs) present in the 3′untranslated region of some specific mRNAs, these proteins function as RNA-binding proteins. The family consists of three members in humans: ZFP36 (also known as tristetraprolin: TTP), ZFP36L1, and ZFP36L2 and one extra member in mice: ZFP36, ZFP36L1, ZFP36L2, and ZFP36L3 (1–4). TTP or ZFP36, the family prototype, physiologically binds to its target mRNAs and stimulates the subsequent target degradation (1, 2). Indeed, ZFP36-deficient mice develop a severe inflammatory disease due mainly to the over-production of tumor necrosis factor-α (TNF-α) by macrophages (5, 6). ZFP36 also directly downregulates the mRNA expression of other proinflammatory cytokines such as IL-2, IL-17, and IFN-γ, which can then modulate T-cell activation and anti-viral immunity (7–10). These mechanistic profiles of ZFP36 raise the possibility that the induction of ZFP36 expression could be an effective therapeutic strategy against inflammatory diseases, particularly in rheumatoid arthritis and other autoimmune diseases in which TNF-α secretion is increased (11, 12).

In contrast to these confirmed functions for ZFP36 as an anti-inflammatory protein, the role of the other ZFP36 family members in the immune system and its particular cells remains largely unknown. Interestingly, lipopolysaccharide (LPS) stimulation in macrophages induces ZFP36 expression, while downregulates the expression of ZFP36L1 and ZFP36L2 (13, 14), suggesting that the expression levels of these three molecules are differently regulated and thus, likely they play different roles. In an attempt to understand the physiological role of ZFP36L1 and ZFP36L2, these genes were knocked out in mice. Lack of ZFP36L1 leads to embryonic lethality at embryonic day 11 in mice due to placental defects (15). In contrast, homozygous *Zfp36l2*-deficient mice are viable; however, they die within ~2 weeks after birth as a consequence of pancytopenia due to hematopoietic stem cell failure (16). Intriguingly, mice carrying double-deficiency of Zfp36l1 and Zfp36l2 in T-cell lineage results in the arrest of thymopoiesis at the double-negative stage and develop T cell acute lymphoblastic leukemia (T-ALL) due to aberrant activation of Notch signaling (17). On the other hand, the single deletion of ZFP36L1 or ZFP36L2 in T-cell lineage does not lead to T-ALL (17). These findings suggest that ZFP36L2 plays a non-redundant role in hematopoietic stem cell differentiation. However, ZFP36L1 and ZFP36L2 have an interconnected role that seems to be redundant during T cell differentiation because the defect in thymopoiesis is observed only when both proteins are simultaneously absent.

Imbalance of T-cell response is present in various autoimmune diseases (18). Because ZFP36L2 is involved in hematopoietic stem cell differentiation and in thymopoiesis, it is not surprising that ZFP36L2 seems to be involved in the development of human autoimmune diseases, particularly in systemic lupus erythematosus (SLE) where *ZFP36L2* was found to be significantly downregulated in peripheral blood mononuclear cells (PBMCs) of SLE patients in comparison to healthy individuals (19). Also, *ZFP36L2* was found to be a disease-susceptibility gene in multiple sclerosis (MS), and decreased *ZFP36L2* expression was observed in MS patients as compared with healthy controls (20). Collectively, these findings suggest that ZFP36L2 is involved in the physiopathology of autoimmune diseases in humans; however, the precise role of ZFP36L2 in a specific T cell population has not been elucidated. Thus, with the goal of better understanding the mechanistic role of ZFP36L2 in autoimmune diseases, we set up experiments to study the expression of ZFP36L2 in CD4^+^ T cells and find novel ZFP36L2-target mRNAs that could modulate regulatory T cells (Tregs). Our results suggest that ZFP36L2 is involved in the suppression function of induced Tregs (iTregs) by accelerating the degradation of *Ikzf2* mRNA.

## Materials and Methods

### Mice

C57BL/6 mice and BALB/c mice were purchased from CLEA (Tokyo, Japan). RAG2^−/−^ mice and Foxp3^YFP−Cre^ mice on a C57BL/6 background were purchased from Jackson Laboratory (Bar Harbor, ME). Foxp3^hCD2^ mice on a BALB/c background were described previously (21). All mice were housed in microisolator cages under specific pathogen-free conditions, and all experiments were performed according to the guidelines of Chiba University established by Chiba University for experiments in animals, which conform to the “Guide for the Care and Use of Laboratory Animals” published by the US National Institutes of Health (NIH Publication, 8th Edition, 2011).

### Reagents

Monoclonal antibodies to murine CD3ε (2C11), CD28 (37.51), CD4 (H129.19), CD44 (IM7), CD62L (MEL-14), CTLA-4 (UC10-4B9), IL-4 (11B11), IFN-γ (XMG1.2), and human NGFR (C40-1475) were purchased from BD Biosciences (San Jose, CA). Monoclonal antibodies to murine Glycoprotein A repetitions predominant (GARP) (F011-5) and Foxp3 (FJK-16s) and anti-mouse/human Helios antibody (22F6) were purchased from eBioscience (San Diego, CA). Anti-latency-associated peptide (LAP) antibody (TW7-16B4) was purchased from BioLegend (San Diego, USA). Human TGF-β was purchased from R&D Systems (Minneapolis, MN).

### Isolation and Stimulation of Human CD4^+^ T Cells

The human subject research component of this study was approved by the Ethics Committee of Chiba University, and written informed consent was obtained according to the Declaration of Helsinki. PBMCs from healthy donors were prepared by using Ficoll-Paque density gradient centrifugation (GE Healthcare, Piscataway, NJ). CD4^+^ T cells were purified from PBMCs with a CD4^+^ T Cell Isolation Kit II (Miltenyi Biotec, Sunnyvale, CA) according to the manufacturer's instructions. The purity of CD4^+^ T cells was routine >98% by FACS analysis. Isolated CD4^+^ T cells (1 × 10^6^ cells/ml) were stimulated with Dynabeads Human T-Activator CD3/CD28 (Thermo Fisher Scientific, Waltham, MA).

### Plasmids

The bicistronic retrovirus vectors used in the experiments [pMX-IRES-hNGFR (pIN), MSCV-IRES-hNGFR (MIN), and MSCV-IRES-GFP (MIG)] have been described previously (22). Expression plasmids of murine ZFP36, ZFP36L1, and ZFP36L2 were kindly provided by Drs. Ching-Jin Chang (National Taiwan University, Taiwan) and Silvia B. V. Ramos. cDNA for *Zfp36, Zfp36l1*, or *Zfp36l2* was subcloned into pIN, MIN, and MIG. cDNA for *Zfp36l1* or *Zfp36l2* was also subcloned into pcDNA3 (Invitrogen, Carlsbad, CA). pGL3-promoter vector (pGL3-pro) was purchased from Promega Biotech, Inc. (Madison, WI). 3′UTR of *Ikzf2* (encoding Helios), which contains three AREs, was cloned into pGL3-pro to construct pGL3-pro-*Ikzf2*-3′UTR (pGL3-pro-*Ikzf2*) by PCR with KOD-Plus-Neo (Toyobo, Osaka, Japan). Primer sequences for cloning of 3′UTR of *Ikzf2* mRNA are as follows: forward, 5′-GCCGTGTAATTCTAGGGCCTTTTCATTCCAAAGGGG-3′, reverse, 5′-GTCTGCTCGAAGCGGTGCTTGTTCCTGAGATTGGCTAAG-3′. Mutants of pGL3-pro-*Ikzf2* lacking each ARE (Del ARE1, Del ARE2, and Del ARE3) were generated using pGL3-pro-*Ikzf2* as a template by Gibson Assembly System (New England BioLabs, Ipswich, MA) according to the manufacturer's instructions. All constructs were verified by sequencing.

### Isolation, Stimulation, and Differentiation of Murine Naïve CD4^+^ T Cells

CD4^+^ CD62L^+^ CD25^−^ TCRαβ^+^ T cells (naïve CD4^+^ T cells) were isolated from spleen using an EasySep™ Mouse Naïve CD4^+^ T Cell Isolation Kit (STEMCELL Technologies, Vancouver, Canada) according to the manufacturer's instructions. The resultant cells were > 98% pure CD4^+^ CD62L^+^ T cells when analyzed by FACS. Cells (1 × 10^6^ cells/ml) were then stimulated with plate-bound anti-CD3 mAb (1 μg/ml) in the presence of anti-CD28 mAb (1 μg/ml) in 48-well plates under neutral conditions [anti-IFN-γ mAb (5 μg/ml) and anti-IL-4 mAb (5 μg/ml)] or iTreg-inducing conditions [TGF-β (1 ng/ml), anti-IFN-γ mAb, and anti-IL-4 mAb].

### RNA-Sequence Analysis

Total RNA was isolated by using an RNeasy Mini Kit (Qiagen, Hilden, Germany). RNA-sequencing was performed as described previously (22). 3′UTR sequences were extracted from the table browser of the UCSC Genome Browser. Genes containing ARE “TATTTAT” in 3′UTR were identified using custom R script. RNA-seq data will be available from the National Center for Biotechnology Information's Gene Expression Omnibus upon publication of the manuscript.

### Quantitative Real-Time PCR Analysis

Total RNA was extracted with Isogen II reagent (Nippon Gene, Tokyo, Japan), and reverse transcription was carried out using an iScript cDNA Synthesis Kit (BioRad, Hercules, CA). Quantitative real-time PCR analysis (qPCR) was performed as described previously (23, 24). The expression levels of each gene were normalized to the levels of β-actin. Sequences of primers are as follows:

Murine *Zfp36* 5′- GTCTCTTCACCAAGGCCATTC-3′,
reverse, 5′- CCGAGTTTATGTTCCAAAGTCC-3′.
Murine *Zfp36l1* 5′- CACGACACACCAGATCCTAGTC-3′,
reverse, 5′- CTGGGAGTGCTGTAGTTGAGC-3′.
Murine *Zfp36l2*, 5′- GGCCGCACAAGCACAAC−3′,
reverse, 5′-GAGACTCGAACCAAGATGAATAACG-3′,
Murine β *-actin*, 5′-CTGTCCCTGTATGCCTCTG-3′,
reverse, 5′-ATGTCACGCACGATTTCC-3′.
Human *ZFP36L2*, 5′-AAACATGTCGACCACACTTCTG-3′,
reverse, 5′-CATGTTGTTCAGGTTGAGGTTG-3′.
Human β*-actin*, 5′-AGGATGCAGAAGGAGATCACTG-3′,
reverse, 5′-GGGTGTAACGCAACTAAGTCATAG-3′.

### Retrovirus-Mediated Gene Expression

The production of retroviruses and infection to murine CD4^+^ T cells was performed as described previously (22–25). In brief, naive CD4^+^ T cells that had been stimulated with plate-bound anti-CD3 mAb/anti-CD28 mAb under neutral conditions for 24 h were added to retrovirus-bound RetroNectin/anti-CD3 mAb-coated plates and were cultured for 24 h. Cells were then stimulated with anti-CD3 mAb/anti-CD28 mAb under neutral conditions or iTreg-inducing conditions for 3 days.

### Intracellular Staining

Intracellular staining of murine CD4^+^ T cells was performed as described previously (22–25) using the following antibodies: anti-Foxp3-FITC (259D/C7, BD Biosciences) and anti-mouse/human Helios-Alexa Fluor^®^ 647 (22F6, BioLegend). Cells were analyzed on a FACS Canto II (Becton Dickinson, San Jose, CA) using FlowJo software (Tree Star, Ashland, OR).

### Decay Rates of *Ikzf2* mRNA

Naïve CD4^+^ T cells of Foxp3^hCD2^ mice were infected with a retrovirus of MIN-*Zfp36l2* or MIN (mock) in iTreg-inducing conditions as described above, and CD4^+^ hCD2^+^ hNGFR^+^ cells (ZFP36L2 iTregs or mock iTregs) were isolated by flow cytometry. These cells were treated with 10 μg/ml of actinomycin D, total RNA was harvested after indicated periods, and mRNA levels of *Ikzf2* were evaluated by qPCR. GraphPad Prism software version 8.12 was used to calculate transcript half-lives based on a one-phase exponential decay model.

### Reporter Assay

EL-4 cells were transfected with either pGL3-pro-*Ikzf2* or ARE deletion mutants of pGL3-pro-*Ikzf2* in the presence of pRL-TK with a Neon Transfection 100 μL kit with buffer R (Thermo Fisher Scientific, Waltham, MA). Where indicated, pcDNA3-*Zfp36l1*, pcDNA3-*Zfp36l2*, or pcDNA3 (as a control) was co-transfected. Four hours later, cells were treated with PMA/Ionomycin for 20 hours. Firefly luciferase activity of the reporters was normalized by Renilla luciferase activity of pRL-TK (Promega Biotech, Inc.).

### RNA Electrophoretic Mobility Shift Assay

The RNA probes were synthesized with the Riboprobe^®^ System-T7 (Promega Biotech, Inc.) using synthetic DNA sequences immediately downstream a T7 promoter, and were body-labeled with [α-^32^P]UTP (3,000 Ci/mmol; Perkin Elmer) during the transcription process as previously described (26, 27). The RNA probes' sequences are *Ikzf2* ARE1 (5′-UUUACUAGGGCUAUUUAUUCCACUAUUU-3′), *Ikzf2* ARE2 (5′-AAGGAUAUUUAUUUCUGAAUGAGGUAAAUAAGUU-3′) and *Ikzf2* ARE3 (5′-UUAUUCAUAUUUAUAUGUAGUGUGUUCU-3′). Protein extracts were prepared from HEK 293 cells transfected with a construct driven by the CMV promoter followed by murine ZFP36L2 or empty vector. The protein extracts were incubated for 15 min at room temperature with 1 x 10^5^ cpm of ^32^P-labeled RNA probes as previously described (26, 27). The resultant reaction mixtures of protein-RNA complexes were then loaded onto 6% non-denaturing acrylamide (37.5:1) gels and subjected to electrophoresis at 150 V for 10 min, followed by electrophoresis at 200 V for 120 min in 0.4 x Tris-borate/EDTA running buffer. The gel was dried, exposed to film (Carestream BIOMAX MR Film), and developed after different times of exposure.

### Suppression Assay

*In vitro* Treg suppression assays were performed as described previously (23, 24) with minor modifications. In brief, naïve CD4^+^ T cells of Foxp3^YFP−Cre^ mice were prepared as described above. After naïve CD4^+^ T cells were infected with a retrovirus of MIN-*Zfp36l2* or MIN (mock) and cultured for 4 days in iTreg-inducing conditions, CD4^+^ YFP^+^ hNGFR^+^ cells (ZFP36L2 iTregs or mock iTregs) were isolated by flow cytometry. Naïve CD4^+^ T cells from WT mice were labeled with carboxyfluorescein diacetate succinimidyl ester (CFSE) and stimulated with soluble anti-CD3 mAb in the presence of irradiated splenocytes for 2 days. Where indicated, ZFP36L2 iTregs or mock iTregs were added at a 4:1 ratio. CFSE dilution profiles of naïve CD4^+^ T cells were assessed by flow cytometry, and data were transformed into Division Index using FlowJo software.

### Adoptive Transfer Model of Colitis

ZFP36L2 iTregs or mock iTregs were prepared as described above and intraperitoneally co-injected (2 × 10^5^ cells/mouse) with naive CD4^+^ T cells (CD45Rb^high^ YFP^−^ CD4^+^ cells from Foxp3^YFP−Cre^ mice, 4 × 10^5^ cells/mouse) to 6–8-week RAG2^−/−^ mice. These mice were weighed twice per week after cell transfer. Moribund mice were euthanized based on Institutional Animal Care and Use Committee protocol. Histopathological scoring was performed as described previously (28) in a blinded fashion.

### Statistical Analysis

Data are summarized as means ± SD. Multiple group comparison was performed by ANOVA followed by Tukey's test. A comparison between 2 groups was analyzed by Student's *t*-test or Chi-squared test. *P* < 0.05 were considered significant.

## Results

### Downregulation of ZFP36L2 Expression in CD4^+^ T Cells by T Cell Receptor (TCR)-Mediated Stimulation

**Figure 1:**
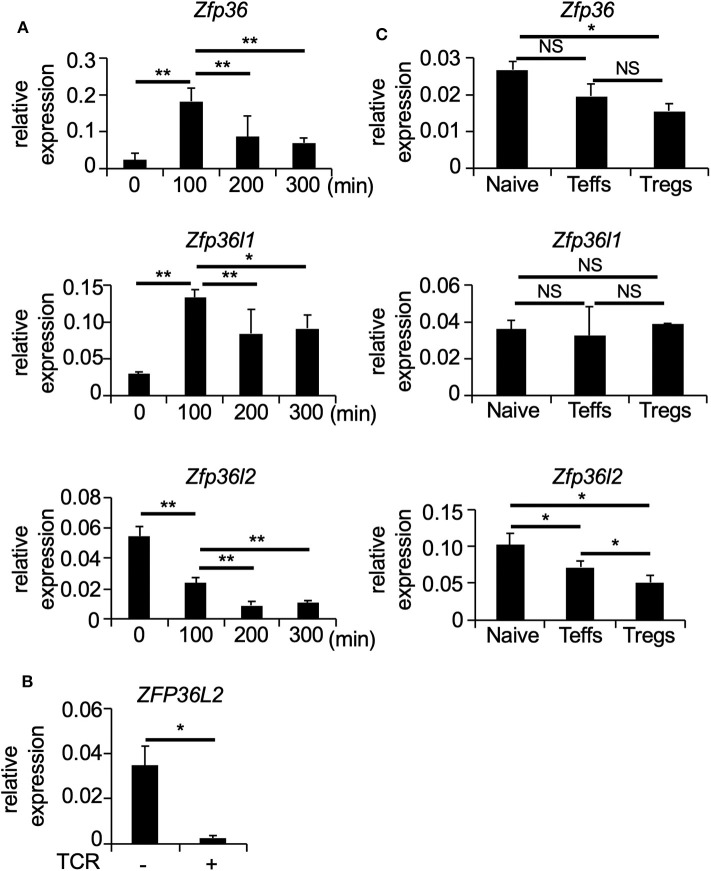
TCR-mediated stimulation downregulates *Zfp36l2* expression. **(A)** Naïve CD4^+^ T cells were isolated from the spleen of C57BL/6 wild-type (WT) mice and stimulated with anti-CD3/anti-CD28 mAb under neutral conditions for indicated periods. Total RNAs were prepared from these cells, and mRNA levels of *Zfp36, Zfp36l1*, and *Zfp36l2* were analyzed by quantitative PCR (qPCR) analysis. Data are means ± SD (*n* = 3, each), representative of three independent experiments. ^*^p < 0.05, ^**^p < 0.005. **(B)** CD4^+^ T cells isolated from PBMCs of healthy donors were stimulated with Dynabeads Human T-Activator CD3/CD28 (TCR+). Freshly isolated CD4^+^ T cells were used as controls (TCR–). Eight hours later, total RNAs were prepared from these cells, and mRNA levels of *ZFP36L2* were analyzed by qPCR analysis. Data are means ± SD (*n* = 3, each). ^*^p < 0.05. **(C)** CD25^−^ CD62L^+^ CD4^+^ T cells (Naive), CD25^−^ CD62L^−^ CD4^+^ T cells (effector CD4^+^ T cells; Teffs), and CD25^+^ CD4^+^ T cells (regulatory CD4^+^ T cells; Tregs) were isolated from spleen of WT mice. Total RNAs were prepared from these cells, and mRNA levels of *Zfp36, Zfp36l1*, and *Zfp36l2* were analyzed by qPCR analysis. Data are means ± SD (*n* = 3, each), representative of two independent experiments. ^*^p < 0.05. NS, not significant.

To determine how T cell receptor (TCR)-mediated stimulation differentially affects the expression of the ZFP36 family members in CD4^+^ T cells, we first isolated naïve CD4^+^ T cells (CD4^+^ CD62L^+^ CD25^−^ TCRαβ^+^ T cells) from spleens of C57BL/6 wild-type (WT) mice and stimulated naïve CD4^+^ T cells with plate-coated anti-CD3 and soluble anti-CD28 antibodies for 100, 200, and 300 min. Then, total RNA was isolated at each time points of TCR-mediated stimulation, and the expression of *Zfp36, Zfp36l1*, and *Zfp36l2* was quantified by qPCR. Consistent with previous studies (10, 29), TCR-mediated stimulation significantly induced *Zfp36* (*p* < 0.005) and *Zfp36l1* (*p* < 0.005) expression, resulting in a 3-fold increase at 100 min and then gradually decreasing thereafter at 200 and 300 min (Figure 1A). In contrast, TCR-mediated stimulation lead to a ~2.5 fold reduction (*p* < 0.005) of the mRNA levels of *Zfp36l2* (Figure 1A). Interestingly, when analogous experiments were done using human CD4^+^ T cells, we also found that the expression of *ZFP36L2* mRNA was reduced by TCR-mediated activation in cells isolated from healthy donors (Figure 1B). Thus, TCR-mediated stimulation resulted in a comparable downregulation of Zfp36l2 in the murine system as well as in humans, suggesting that the mechanism for this downregulation is conserved in both species. However, differentiation of naïve CD4^+^ T cells into effector T cells (Teffs) or Tregs *in vivo* requires other factors that could potentially affect the expression pattern of ZFP36 family members. So, to test if these genes behaved similarly when CD4^+^ T cells had been *in vivo* differentiated, we isolated three populations of CD4^+^ T cells from WT mice: (i) CD4^+^ CD62L^+^ CD25^−^ TCRαβ^+^ T cells, namely naïve CD4^+^ T cells; (ii) CD4^+^ CD62L^−^ CD25^−^ T cells, referred as Teffs; and (iii) CD4^+^ CD25^+^ T cells, known as Tregs. We found that mRNA levels of *Zfp36l2* were significantly lower in Tregs when compared to those in naïve CD4^+^ T cells or Teffs (Figure 1C). The levels of *Zfp36* mRNA in naïve CD4^+^ T cells and Teffs were not significantly different; however, Tregs cells expressed significantly lower levels of *Zfp36* mRNA than in naïve CD4^+^ T cells (Figure 1C). On the other hand, mRNA levels of *Zfp36l1* were relatively similar among naïve CD4^+^ T cells, Teffs, and Tregs. These results indicate that the expression pattern of ZFP36L2 is regulated differently than ZFP36 or ZFP36L1, particularly in Tregs, suggesting that the lower expression of ZFP36L2 could be meaningful in Treg function.

### ZFP36L2 Suppresses *Ikzf2* (Encoding Helios) Expression in iTregs

Because ZFP36L2 can bind and stimulate the degradation of ARE-containing mRNAs (17, 26, 30), we raised the possibility that ZFP36L2 could alter the development and function of Tregs by modulating some specific mRNAs. To determine which target mRNAs were potentially subjected to ZFP36L2 modulation, we developed a system in which ZFP36L2 expression was comprehensively higher than the normal physiological levels. So, we could search for ARE-containing mRNAs that were downregulated when the expression of ZFP36L2 was artificially forced. Naïve CD4^+^ T cells were infected with a retrovirus of MIG-*Zfp36l2* or with a control retrovirus MIG (mock). After infection, these cells were cultured under neutral conditions for three additional days, and then based on the simultaneous expression of CD4 and GFP by the retrovirus infection, the cells were sorted by flow cytometry. Two populations of cells were isolated: (i) one in which ZFP36L2 was highly expressed due to the retroviral infection and (ii) another “mock” retroviral infection, in which ZFP36L2 expression was derived only from the endogenous gene. Total RNA was extracted from both cell populations, and gene expression of these cells was evaluated by RNA-seq analysis.

**Figure 2:**
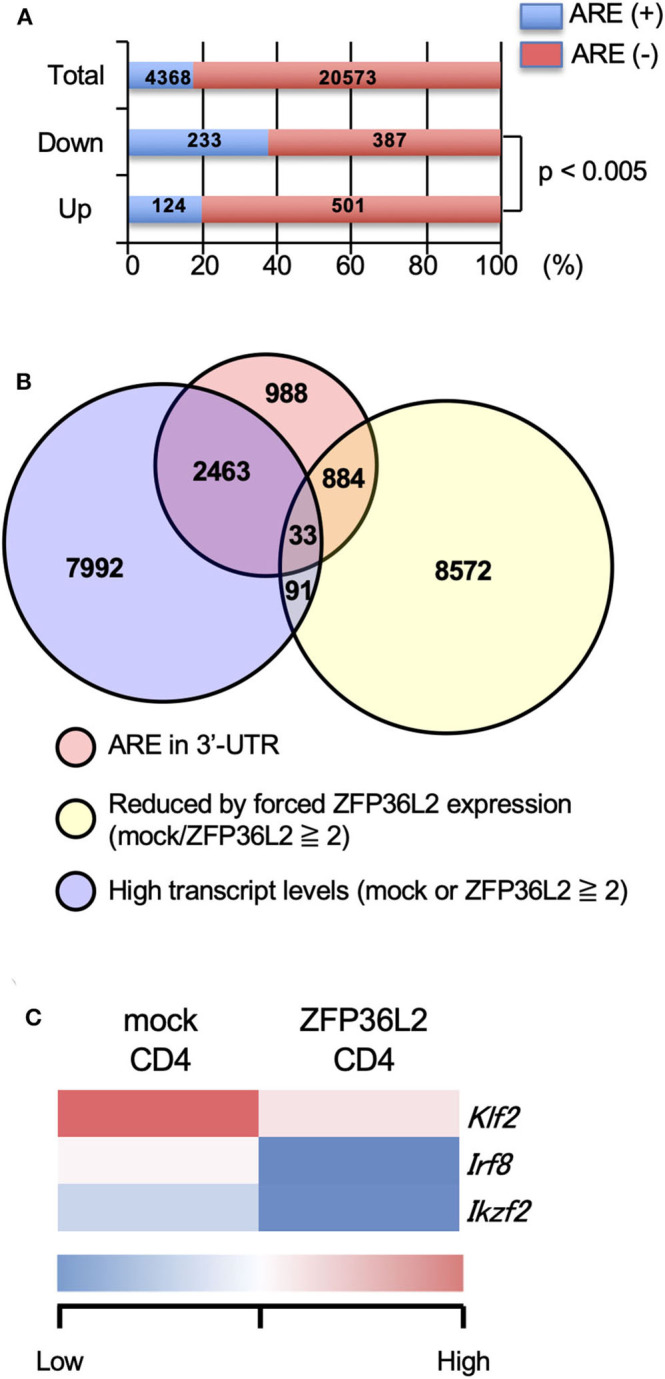
*Ikzf2* (encoding Helios) is downregulated by forced ZFP36L2 expression. **(A–C)** After naïve CD4^+^ T cells were stimulated with anti-CD3/anti-CD28 mAb under neutral conditions for 24 h, cells were infected with retroviruses of MSCV-IRES-GFP-*Zfp36l2* (MIG-*Zfp36l2*) or MIG, and cultured under neutral conditions. Three days later, GFP^+^ cells from MIG-*Zfp36l2*-infected CD4^+^ T cells and from MIG-infected CD4^+^ T cells were isolated by flow cytometry. Total RNAs were prepared from these cells and subjected to RNA-seq analysis. **(A)** Quantification of mRNAs with or without AREs in 3′UTR of mRNA downregulated (Down) or upregulated (Up) in CD4^+^ T cells by ZFP36L2 expression (transcript levels ≧2, fold change ≧1.5). **(B)** Venn diagram showing the overlap of indicated criteria in mRNAs expressed in CD4^+^ T cells. **(C)** Shown is a heat map of differentially expressed genes encoding transcriptional factors among 33 genes enriched in **(B)**.

Initially, we evaluated the presence or absence of AREs in the 3′UTR of all detected genes by the RNA-seq analysis and found that 4,368 over 24,941 (17.5%) contained an ARE (Figure 2A). Upregulated genes and downregulated genes were defined as: [1] Expression levels ≥2 FPKM (fragments per kilobase of exon per million mapped fragments) in mock-transfected CD4^+^ T cells or ZFP36L2-transfected CD4^+^ T cells, and [2] FPKM changed ≥1.5-fold by forced ZFP36L2 expression. As expected, genes containing AREs in 3′UTR were significantly enriched in the downregulated genes when ZFP36L2 was overexpressed; 233 over 620 downregulated genes contained an ARE sequence (37.6%) (Figure 2A). We next identified 33 candidate genes in CD4^+^ T cells that simultaneously fulfill three criteria (Figure 2B): [1] Expression level (FPKM ≥ 2) in mock-transfected CD4^+^ T cells or ZFP36L2-transfected CD4^+^ T cells; [2] FPKM was reduced ≥2-fold by forced ZFP36L2 expression; [3] Gene contains an ARE sequence in the 3′UTR. Among these 33 genes that satisfied the above three criteria (Figure S1), we then searched for transcription factors that could influence CD4^+^ T cell differentiation, and we found three transcription factors (*Klf2, Irf8*, and *Ikzf2*) (Figure 2C). We decided to focus on *Ikzf2*, which encodes Helios, because *Ikzf2* has been described to be involved in Treg function and differentiation (24, 31).

**Figure 3:**
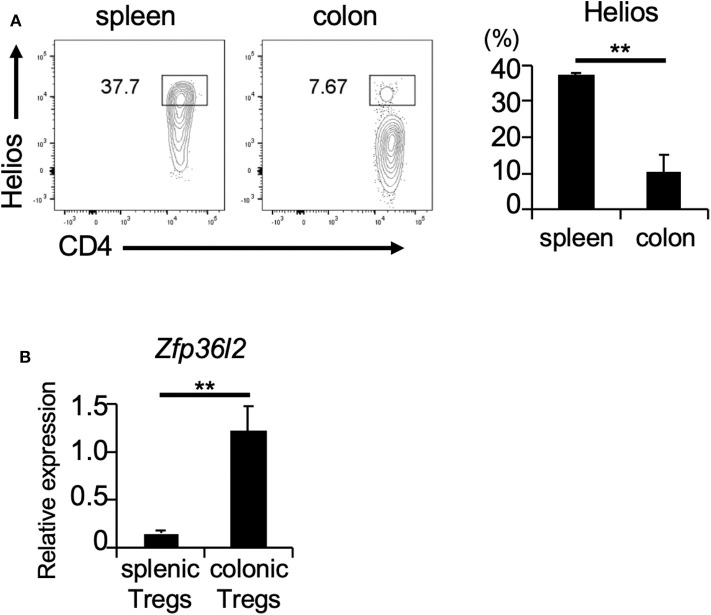
*Zfp36l2* expression is inversely correlated with Helios expression in Tregs. **(A)** CD4^+^ YFP^+^ Tregs were isolated from the spleen and colon of Foxp3^YFP−Cre^ mice. Representative CD4 vs. Helios staining of CD4^+^ YFP^+^ cells and the frequency of CD4^+^ Helios^+^ cells are shown. Data are means ± SD (*n* = 3, each), ^**^p < 0.005, representative of two independent experiments. **(B)** Total RNAs were prepared from CD4^+^ YFP^+^ cells in spleen and colon of Foxp3^YFP−Cre^ mice, and mRNA levels of *Zfp36l2* were analyzed by qPCR analysis. Data are means ± SD (*n* = 3, each), ^**^p < 0.005, representative of two independent experiments.

If ZFP36L2 regulates the Helios expression *in vivo*, one would expect that increased expression levels of ZFP36L2 would result in lower Helios levels and vice-versa. Thus, we set up experiments to investigate the correlation between ZFP36L2 and Helios expression in murine Tregs *in vivo*. Foxp3 is a well-accepted marker of Tregs (32); thus, we used Foxp3^YFP−Cre^ mice as a strategy to isolate Tregs by sorting YFP-expressing cells by flow cytometry. We isolated two distinct T cell populations from Foxp3^YFP−Cre^ mice: (i) CD4^+^ YFP^+^ cells in the spleen [splenic Tregs], which mainly consist of thymus-derived Tregs and (ii) CD4^+^ YFP^+^ cells in the large intestine [colonic Tregs], which mainly consist of peripherally induced Tregs. We observed that the frequency of Helios-expressing cells was significantly higher in splenic Tregs (37.7%) than in colonic Tregs (7.67%) (*p* < 0.005) (Figure 3A), consistent with a previous report (33). Then, we used these two Treg populations to evaluate the expression of *Zfp36l2* mRNA. Intriguingly, *Zfp36l2* mRNA levels were significantly higher in colonic Tregs, which express low levels of Helios, than those in splenic Tregs, which express high levels of Helios (Figure 3B). These observations raise the possibility that *Zfp36l2* would need to be downregulated for thymus-derived Treg differentiation, allowing high expression levels of Helios protein in this population. Conversely, to favor peripherally induced Treg differentiation, *Zfp36l2* would need to be upregulated, consequently resulting in low levels of Helios protein.

**Figure 4:**
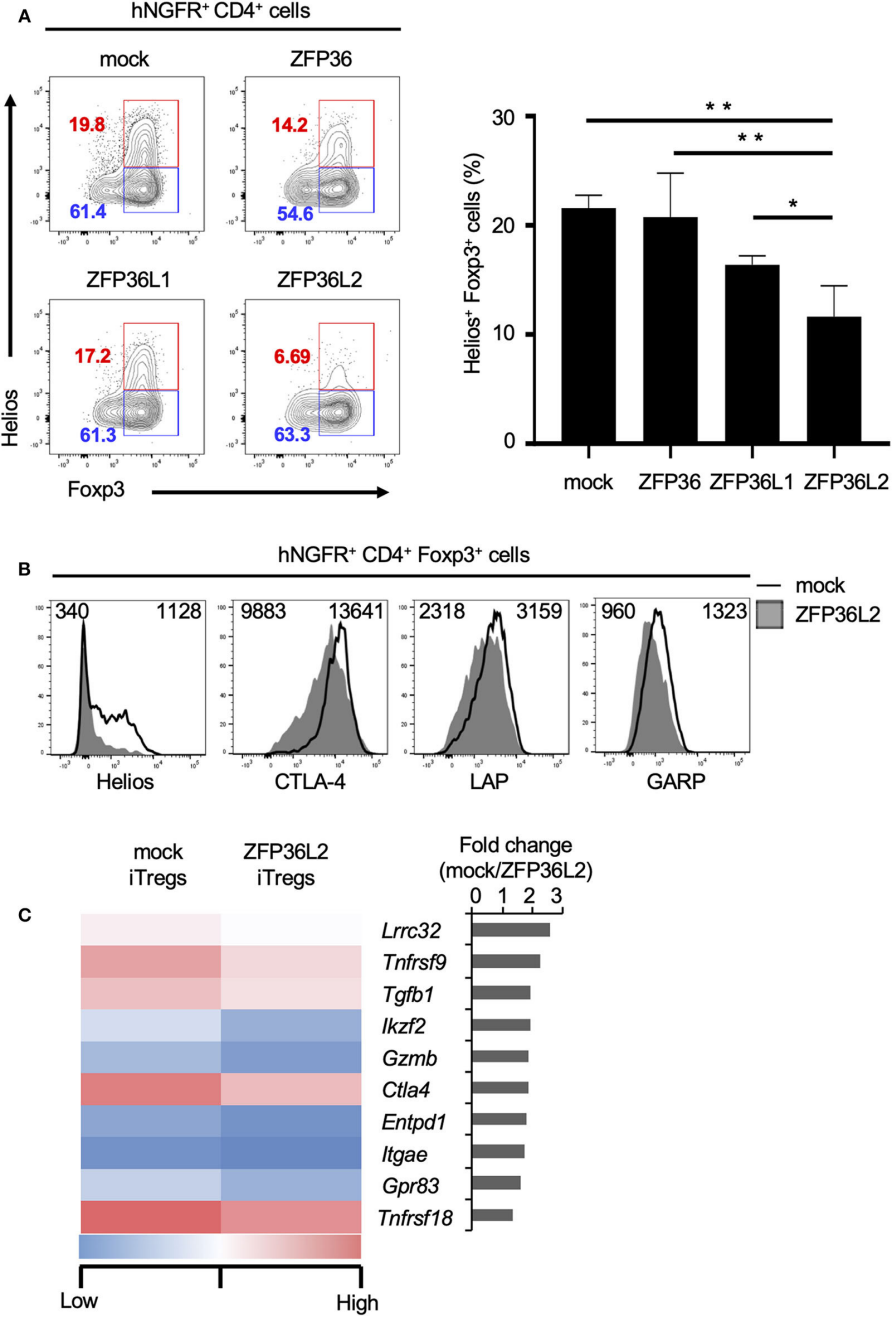
ZFP36L2 inhibits the development of Helios^+^ iTregs. **(A,B)** Naïve CD4^+^ T cells from BALB/c mice were stimulated with anti-CD3/anti-CD28 mAb under neutral conditions for 24 h, infected with retroviruses of MSCV-IRES-hNGFR (MIN)-*Zfp36*, MIN-*Zfp36l1*, MIN-*Zfp36l2*, or MIN (mock), and cultured under iTreg-inducing conditions. **(A)** The expression of Foxp3 and Helios in hNGFR^+^ CD4^+^ cells was analyzed. Shown are representative Foxp3 vs. Helios staining of hNGFR^+^ CD4^+^ cells and means ± SD of the percentages of Helios^+^ Foxp3^+^ cells *n* = 3. **(B)** The expression of indicated molecules in hNGFR^+^ CD4^+^ Foxp3^+^ cells were analyzed by flow cytometry. Shown are representative histograms with mean fluorescence intensity *n* = 3. **(C)** After naïve CD4^+^ T cells of Foxp3^hCD2^ mice were stimulated with anti-CD3/anti-CD28 mAb under neutral conditions for 24 h, cells were infected with retroviruses of MIG-*Zfp36l2* or MIG, and cultured under iTreg-inducing conditions. Three days later, GFP^+^ hCD2^+^ cells from MIG-*Zfp36l2*-infected CD4^+^ T cells (ZFP36L2 iTregs) and from MIG-infected CD4^+^ T cells (mock iTregs) were isolated by flow cytometry. Total RNAs were prepared from these cells and subjected to RNA-seq analysis. Shown is a heat map of differentially expressed genes encoding Treg-associated molecules between Mock-iTregs and ZFP36L2-iTregs. ^*^p < 0.05, ^**^p < 0.005.

To test if any ZFP36 family members or only ZFP36L2 could modulate Helios proteins and iTreg differentiation, we used the retrovirus expression system to express each ZFP family member in naïve CD4^+^ T cells and examined the effect of each ZFP36 family member on Foxp3 and Helios expression in CD4^+^ T cells under iTreg-inducing conditions. Intracellular staining of Foxp3 and Helios in CD4^+^ T cells revealed that the percentage of Helios^−^ iTregs (Helios^−^ CD4^+^ Foxp3^+^ cells) among the total iTreg population was not significantly affected by the expression of ZFP36L2 in comparison to mock transfection, 61.4% vs. 63.3%, respectively (Figure 4A). In contrast, the percentage of Helios-expressing CD4^+^ Foxp3^+^ cells was markedly decreased when ZFP36L2 was expressed in CD4^+^ T cells compared to mock-transfected CD4^+^ T cells (Figure 4A). As suspected, forced expression of ZFP36 or ZFP36L1 did not significantly affect the frequency of Helios-expressing CD4^+^ Foxp3^+^ cells (Figure 4A). These findings suggest that ZFP36L2, but not ZFP or ZFP36L1, can modulate *Ikzf2* mRNA and Helios protein expression in CD4^+^ T cells induced to differentiate into Helios-expressing iTregs.

Also, we have previously shown that Helios effectively upregulates the expression of other molecules present in iTregs (24), such as CD103 and Glucocorticoid-induced TNFR-related protein (GITR). Particularly, Helios-expressing iTregs express higher mRNA levels of glycoprotein A repetitions predominant (GARP), folate receptor 4 (FR4), and G protein-coupled receptor 83 (GPR83) than in control iTregs (24). Because we observed that ZFP36L2 decreases the subpopulation of iTregs expressing Helios as well as the levels of Helios expression, we decided to investigate if the expression of these other molecules associated with Helios presence in iTregs were also affected by the forced expression of ZFP36L2. As expected, ZFP36L2-transfected iTregs expressed markedly reduced Helios levels when compared with mock-transfected control iTregs (Figure 4B). However, we observed only a modest decrease of cytotoxic T lymphocyte-associated antigen 4 (CTLA-4), LAP, and GARP expression levels. Subsequently, with the goal of broadly investigate the consequences of forced expression of Zfp36l2 in reducing gene expression of other Treg-associated molecules, we decided to use these two populations of cells, mock and ZFP36L2-transfected iTregs to isolate mRNAs and perform RNA-seq analysis. This allowed us to investigate other Treg-associated molecules, such as *Lrrc32, Tnfrsf9*, and *Tgfb1*, which encodes GARP, CD137/4-1BB, and TGF-β1, respectively. In Figure 4C, we summarized the top 10 Treg-associated genes that were found to be downregulated when ZFP36L2 was overexpressed. These results suggest that ZFP36L2 reduces the expression of Helios and successively some other Treg-associated molecules in iTregs.

### ZFP36L2 Destabilizes 3′UTR of *Ikzf2* mRNA

**Figure 5:**
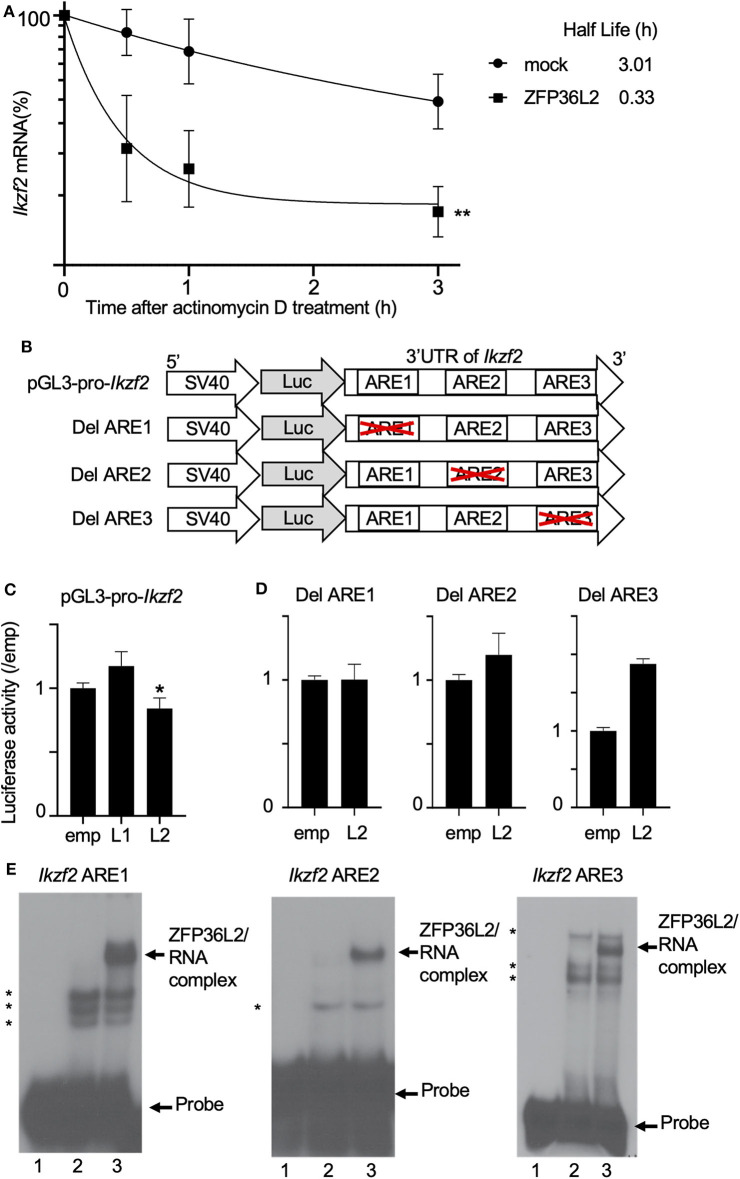
ZFP36L2 destabilizes 3′UTR of *Ikzf2* mRNA. **(A)** Kinetics of *Ikzf2* mRNA levels in sorted mock- or ZFP36L2-transfected iTregs after actinomycin D treatment. The amount of *Ikzf2* mRNA at 0 h was assigned 100%. Trendlines (Graphpad Prism) were fitted to predict mRNA half-lives. Data are means ± SD (*n* = 3, each), ^**^p < 0.005, representative of two independent experiments. **(B)** Schematic diagram of reporter constructs of pGL3-pro-*Ikzf2*-3′UTR (pGL3-pro-*Ikzf2*), and ARE deletion mutants. **(C,D)** EL-4 cells were transfected with pRL-TK and pGL3-pro-*Ikzf2* in the presence of either pcDNA3-*Zfp36l1* (L1), pcDNA3-*Zfp36l2* (L2), or pcDNA3 (emp) (as a control) **(C)**. EL-4 cells were transfected with pRL-TK and either Del ARE1, Del ARE2, or Del ARE3 in the presence of pcDNA3-*Zfp36l2*(L2) or pcDNA3 (emp) **(D)**. Four hours later, cells were stimulated with PMA/Iono for 20 h. Luciferase activity was measured by the dual-luciferase reporter system. Data are means ± SD (*n* = 3, each), ^*^p < 0.05, representative of two independent experiments. **(E)** RNA electrophoretic mobility shift assays were performed by incubating protein extracts from HEK 293 cells transfected with either an empty vector (10 μg, lanes 2) or a vector expressing ZFP36L2 (10 μg, lanes 3) with each *Ikzf2* ARE probe, as indicated on top of the lanes. Lanes 1 in all three panels contain each probe without the addition of any protein extracts. ZFP36L2 caused a shift in the electrophoretic mobility of all three ARE probes (arrow). Note that when protein extracts lacking ZFP36L2 protein (lanes 2) were incubated with each probe, other bands appeared (indicated by asterisks). They correspond to complexes of proteins present in HEK 293 cells that interact with the probes.

We suspected that ZFP36L2 is downregulating the Helios expression in CD4^+^ T cells by binding to *Ikzf2* mRNA, which triggers the destabilization of this mRNA, consequently leading to low expression levels of Helios protein. To investigate whether ZFP36L2 affects mRNA stability of *Ikzf2* in iTregs, we measured the decay rate of *Ikzf2* mRNA in sorted ZFP36L2- or mock-transfected iTregs treated with actinomycin D. We observed that *Ikzf2* mRNA had a shorter half-life in ZFP36L2-transfected iTregs (t_1/2_ > 0.33 h) than that in mock-transfected iTregs (t_1/2_ > 3.01 h) (Figure 5A).

Because the binding of ZFP36L2 to a particular target mRNA is dependent on the presence of ARE sequences in 3′UTR, we decided to investigate if the ZFP36L2 downregulation effect was dependent on the 3′UTR sequence of *Ikzf2* mRNA. As illustrated in Figure 5B, we created a reporter construct in which the 3′UTR of *Ikzf2* mRNA was inserted just after the coding sequence of the luciferase gene of pGL3-pro (pGL3-pro-*Ikzf2*). Three putative AREs were found to be present in the 3′UTR of the mouse *Ikzf2* transcript. In order to investigate the contribution of each of these AREs on the destabilization of this transcript, we created mutant reporter constructs in which individual AREs were deleted (Del ARE1, Del ARE2, and Del ARE3, schematically represented in Figure 5B). As shown in Figure 5C, the luciferase activity of pGL3-pro-*Ikzf2* was decreased by the expression of ZFP36L2 but not by ZFP36L1. Deletion of ARE1 or ARE2 did not significantly affect the luciferase activity in the presence of ZFP36L2 (Figure 5D), suggesting mild contribution of these AREs on the ZFP36L2-mediated downregulation of *Ikzf2* in this experimental setting. Intriguingly, the deletion of ARE3 resulted in increased luciferase activity, although the reason remains unknown.

Additionally, to test if ZFP36L2 could bind directly to these AREs located at 3′UTR of *Ikzf2* transcript, we designed three different probes *Ikzf2* ARE1, ARE2, and ARE3, each containing only one ARE. We then performed RNA gel shift assays with these probes using protein extracts overexpressing or lacking ZFP36L2. As illustrated in Figure 5E, ZFP36L2 prominently bound to each of these three probes (arrows in lane 3 of each panel). These results confirm a direct biochemical interaction between ZFP36L2 protein and each of these individual AREs of the *Ikzf2* mRNA, confirming that ZFP36L2-mediated downregulation of Helios expression is dependent on specific ARE sequences present at the 3′UTR of *Ikzf2* mRNA.

### ZFP36L2 Inhibits the Ability of iTregs

**Figure 6:**
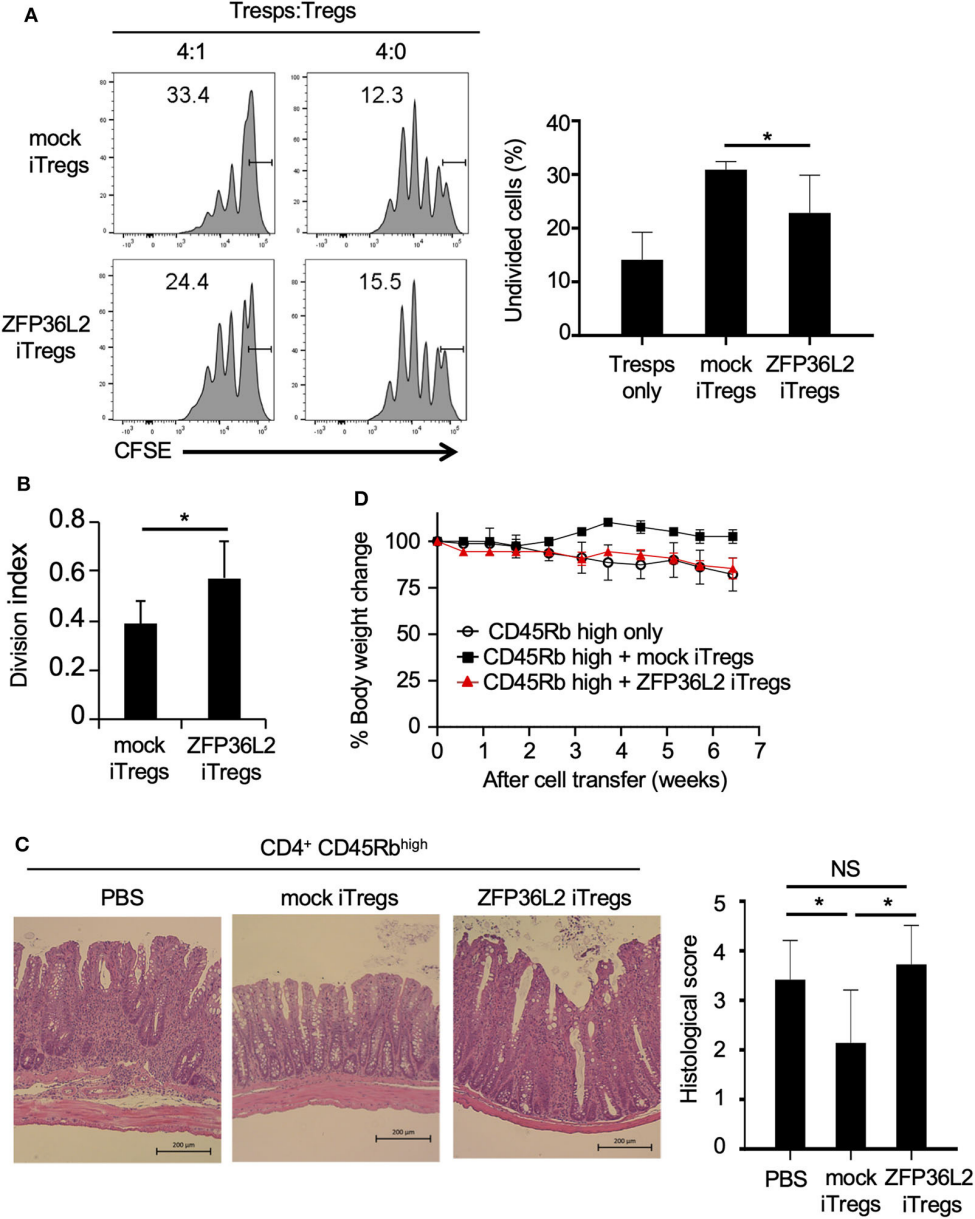
ZFP36L2 inhibits the suppression function of iTregs. **(A,B)** YFP^+^ hNGFR^+^ cells from MIN-*Zfp36l2*-infected CD4^+^ T cells (ZFP36L2 iTregs) and those from MIN-infected CD4^+^ T cells (mock iTregs) of Foxp3^YFP−Cre^ mice were prepared as described in the Materials and Methods. Naïve CD4^+^ T cells were labeled with CFSE and stimulated with soluble anti-CD3 mAb in the presence of irradiated splenocytes. Where indicated, ZFP36L2 iTregs or mock iTregs were added at a 4:1 ratio. CFSE dilution profiles of naïve CD4^+^ T cells were assessed by flow cytometry. **(A)** Representative histograms of CFSE dilution and means ± SD of the percentages of undivided cells. **(B)** Division index using FlowJo software. Means ± SD (*n* = 6, each), ^*^p < 0.05. Similar results were obtained in two independent experiments. **(C,D)** Naive CD4^+^ T cells (CD45Rb^high^ YFP^−^ CD4^+^ cells from Foxp3^YFP−Cre^ mice) were injected into RAG2^−/−^ mice to induce colitis. Where indicated, pMX-IN-*Zfp36l2*-transfected iTreg (ZFP36L2 iTregs) or pMX-IN-transfected iTregs (mock iTregs) were co-injected to the mice. **(C)** Shown are representative H&E stained sections of the colon and means ± SD of histopathological scores at 7 weeks after the cell transfer. ^*^p < 0.05, bar: 200 μm. **(D)** Shown are changes in body weight after the cell transfer (three mice in each group).

iTregs can inhibit the proliferation of naïve CD4^+^ T cells (33); however, it is not known if iTregs, which preferentially differentiate into Helios-low iTregs by forced expression of ZFP36L2, retain their ability to inhibit naïve CD4^+^ T cell proliferation. To address this question, naive CD4^+^ T cells isolated from Foxp3^YFP−Cre^ mice were infected with a retrovirus of MIN-*Zfp36l2* or MIN (as a control) and cultured for an additional 4 days in iTreg-inducing conditions. These cultured cells were subjected to flow cytometry to isolate only the retrovirus-infected iTregs (CD4^+^ YFP^+^ hNGFR^+^ cells), which were then co-cultured under neutral conditions with freshly isolated naïve CD4^+^ T cells (responder T cells:Tresps) to evaluate the ability of these iTregs to inhibit the proliferation of Tresps. iTregs overexpressing ZFP36L2 (ZFP36L2 iTregs) exhibit significantly weak ability to suppress Tresp proliferation than the control iTregs (mock iTregs), which suppress Tresp proliferation (Figures 6A,B).

We finally examined the function of ZFP36L2 iTregs *in vivo* by using a T cell transfer model of colitis (28). In this experiment, ZFP36L2 iTregs or mock iTregs were adoptively co-injected with naive CD4^+^ T cells into RAG2^−/−^ mice, and the development of colitis was evaluated by histological analyses and by its effect on the mice weight. In the absence of iTreg injection, recipient RAG2^−/−^ mice developed colitis (Figure 6C) and reduced body weight (Figure 6D, open circles). Notably, when the mice were co-injected with mock iTregs, the histological scores improved, and there was significant body weight gain (Figures 6C,D). However, when ZFP36L2 iTregs were co-injected, no significant improvement was observed on histological evaluation of the colitis nor the body weight, suggesting that ZFP36L2 iTregs have limited suppression function *in vivo*.

## Discussion

Imbalance of the immunological self-tolerance response is the basis of several autoimmune diseases (18). One of the critical cell populations in building up an effective immunological self-tolerant response is Tregs. These cells are particularly important in maintaining the induced tolerance to peripheral self-antigens as well as suppressing deleterious immune responses against the host itself. In this study, we focused on the role of the RNA-binding protein ZFP36L2 in modulating the differentiation and function of Tregs. Initially, we observed that TCR-mediated stimulation downregulates ZFP36L2 expression in human and murine CD4^+^ T cells (Figures 1A,B). Additionally, we found that ZFP36L2 expression levels were lower in Tregs than those in naïve CD4^+^ T cells (Figure 1C), suggesting that during the differentiation process of naïve CD4^+^ T cells into iTregs, TCR stimulation may negatively modulate the ZFP36L2 expression. In contrast, when we artificially forced ZFP36L2 expression, it inhibited the expression of several transcription factors, particularly Helios. The differentiation of iTregs in the presence of ZFP36L2 resulted in the differentiation of iTregs, preferentially not expressing Helios (Figure 4). The mechanism by which ZFP36L2 is downregulating Helios expression is likely to be the induction of *Ikzf2* mRNA degradation since ZFP36L2 action is dependent on the endogenous 3′UTR sequence of *Ikzf2* mRNA which contains ARE-binding elements (Figure 5). Interestingly, ZFP36L2-expressing iTregs have reduced suppression property on the proliferation of naïve CD4^+^ T cells *in vitro* as well as the development of the T cell transfer model of colitis (Figure 6). These results suggest that the reduction of ZFP36L2 expression during Treg differentiation boosts the preferential differentiation of Helios^+^ iTregs, which are known to efficiently suppress the proliferation of naïve CD4^+^ T cells, thus maintaining a more effective regulatory function over activated CD4^+^ T cells.

We found that in contrast to the other ZFP36 family members, ZFP36 and ZFP36L1, ZFP36L2 is expressed to a greater degree in naïve CD4^+^ T cells than in Teffs, whereas the expression of ZFP36 or ZFP36L1 in naïve CD4^+^ T cells was similar to that in Teffs (Figure 1C). We also found that TCR-mediated stimulation decreased ZFP36L2 expression but increased the expression of ZFP36 and ZFP36L1 in CD4^+^ T cells (Figures 1A,B). These findings were largely consistent with recent reports (10, 29). Moore et al. have shown that ZFP36 and ZFP36L1 were induced in CD4^+^ T cells upon activation (10), and Salerno et al. have demonstrated that the expression of ZFP36L2 mRNAs in CD4^+^ T cells was decreased by PMA/Ionomycin stimulation (29). We also found that genes that are known to be highly expressed in naïve CD4^+^ T cells and downregulated by TCR-mediated activation, including *Stat5a* and *Tnfrsf10b*, were upregulated by the forced expression of ZFP36L2 in CD4^+^ T cells (data not shown). These findings raise the possibility that the ZFP36L2 could be involved in maintaining CD4^+^ T cells in a naïve state.

Our findings suggest that maintaining a low ZFP36L2 expression level is important for maintaining Treg function *in vivo*. We found that the expression levels of ZFP36L2 in Tregs were significantly lower than those in Teffs or naïve CD4^+^ T cells (Figure 1C). We also found that the forced expression of ZFP36L2 decreased the suppression function of Tregs in the *in vitro* suppression assay as well as in the adoptive transfer model of colitis (Figure 6). Consistently, the forced expression of ZFP36L2 decreased the expression of Treg-associated molecules, including CTLA-4, LAP, and GARP, as well as Helios in iTregs (Figure 4B). In this regard, because 3′UTR of mRNAs of *Ctla4, Tgfb1*, or *Lrrc32* does not contain AREs for binding of ZFP36L2 (data not shown), it is possible that ZFP36L2-mediated reduction of these Treg-associated molecules is achieved through indirect mechanisms rather than direct degradation of their mRNAs.

We showed that ZFP36L2 reduces Helios expression in CD4^+^ T cells under iTreg-inducing conditions. Regarding the roles of Helios in Treg function, it was initially reported that mice with a T cell-specific deletion of Helios appear to have normal Treg function (34). However, recent reports have shown that Foxp3-specific-Helios-deficient mice, as well as systemic Helios-deficient mice, spontaneously develop delayed-onset autoimmune-like conditions with multi-organ inflammation, systemic lymphocyte activation, and anti-nuclear and anti-DNA antibodies (35, 36). In addition, we have previously shown that the forced expression of Helios induces the expression of the Treg–related molecules GITR, CD103, GARP, and FR4 and enhances the suppression function in murine Tregs (24). Furthermore, it has been shown that Helios deficiency results in the unstable phenotypes in Tregs during inflammatory responses due to the production of proinflammatory cytokines, including IFN-γ, IL-17, and TNF-α (35–37). Moreover, mice with Helios-deficiency in Tregs exhibited enhanced antitumor immunity (36). Given that ZFP36L2 induction in CD4^+^ T cells profoundly reduced Helios expression with a modest reduction of other Treg-related molecules (Figure 4), we speculate that the fine-tuning of ZFP36L2 expression in Tregs is necessary for appropriate Helios expression and the function of Tregs.

Helios expression has been shown to be regulated in several ways. We have previously demonstrated that Helios is induced by TGF-β and suppressed by IL-6/STAT3 signaling in CD4^+^ T cells (24) and that cell-intrinsic Foxp3 expression is essential for Helios induction in murine iTregs (24). Yet numerous other explanations have been discovered for Helios induction and maintenance. Recent studies have shown that IL-2/STAT5 signaling pathway promotes Helios induction in Tregs in humans and mice (38, 39). Vitamin D receptor-mediated signaling has been demonstrated to activate *Ikzf2* gene transcription in Tregs through vitamin D-responsive elements (40). On the other hand, Nakagawa et al. reported that activation of GITR using the agonistic anti-GITR antibody DTA-1 decreases Helios expression in Tregs and favors their conversion to Teffs *in vivo* (36). We demonstrated here that ZFP36L2 markedly inhibited Helios expression (Figure 4) and that 3′UTR of *Ikzf2* mRNA seems to be a target of ZFP36L2-mediated destabilization (Figure 5). These findings suggest that Helios expression seems to be regulated by several distinct mechanisms in CD4^+^ T cells whose relative importance should be determined in future research.

Among ZFP36 family members, we found that ZFP36L2 but not ZFP36 or ZFP36L1 suppressed the expression of Helios in CD4^+^ T cells under iTreg-inducing conditions (Figure 4A). Also, the expression of these three members was differently regulated in CD4^+^ T cells (Figure 1A). These findings suggest that the roles of ZFP36 family members in Tregs differ from each other. On the other hand, Hodson et al. have shown that mice lacking both ZFP36L2 and ZFP36L1 but not either ZFP36L2 or ZFP36L1 in lymphoid lineages develop leukemia (17), suggesting some redundant roles.

Interestingly, Salerno et al. have recently reported that ZFP36L2 represses IFN-γ production in CD44^hi^ CD4^+^ T cells at the translational level rather than effecting the mRNA stability (29). We showed here that ZFP36L2 suppresses iTreg function. Based on these findings, we suppose that ZFP36L2′s roles in CD4^+^ T cells seem to differ depending on the cell type. It is also possible that these different ZFP36L2 mechanisms may influence and complement each other *in vivo*. Indeed, the suppression of IFN-γ production by ZFP36L2 may partly influence the results of the colitis model because IFN-γ is well-known to be involved in the induction of colitis. Further studies, including specific deletion of ZFP36 family members in Tregs, are needed to elucidate the precise roles of ZFP36 family members in Helios expression and Treg suppression.

In conclusion, we show that ZFP36L2 suppresses iTreg function, possibly by destabilizing *Ikzf2* mRNA. Although further studies are needed, selective fine-tuning of ZFP36L2 affecting Helios expression in iTregs could be an effective strategy for the treatment of autoimmune diseases.

## Data Availability Statement

The datasets generated for this study can be found in the National Center for Biotechnology Information's Gene Expression Omnibus (GSE150937) upon publication of the article.

## Ethics Statement

The studies involving human participants were reviewed and approved by Ethics Committee of Chiba University. The patients/participants provided their written informed consent to participate in this study. The animal study was reviewed and approved by Chiba University for experiments in animals.

## Author Contributions

HT has full access to all data generated by this study and takes responsibility for the data and also designed and performed the experiments and wrote the manuscript. SM performed the experiments, prepared reagents, and wrote the manuscript. AI analyzed the data of RNA-seq analyses. ST, AS, and KS proposed the projects and designed the experiments. SF and KI proposed the projects. SR provided reagents, helped design constructs, performed and interpreted experiments, and wrote the manuscript. HN proposed the projects, designed the experiments, and wrote the manuscript. All authors were involved in drafting the manuscript and approved the final version to be published.

## Conflict of Interest

The authors declare that the research was conducted in the absence of any commercial or financial relationships that could be construed as a potential conflict of interest.

## Supplementary Material

Figure S1 Genes whose expression is downregulated by the forced expression of ZFP36L2. Shown is a heat map of the 33 differentially expressed mRNAs described in Figure 2B.

## Abbreviations

ARE: AU-rich element
CTLA-4: cytotoxic T lymphocyte-associated protein 4
Foxp3: forkhead box p3
FPKM: fragments per kilobase of exon per million mapped fragments
GARP: glycoprotein-A repetitions predominant
Ikzf2: Ikaros family zinc finger 2
iTregs: induced Tregs
LAP: latency-associated peptide
MIG: MSCV-IRES-GFP
MIN: MSCV-IRES-hNGFR
PBMC: peripheral blood mononuclear cells
qPCR: quantitative real-time PCR
Teffs: effector T cells
Tregs: regulatory T cells
Zfp36l1, zinc finger protein 36: CCCH type-like 1
Zfp36l2, zinc finger protein 36: CCCH type-like 2.
