# Supplementary figures and images for: RNA-Binding Protein ZFP36L2 Downregulates Helios Expression and Suppresses the Function of Regulatory T Cells
Source: Front Immunol. 2020 Jun 23;11:1291. doi: 10.3389/fimmu.2020.01291 (PMC7324482; doi:10.3389/fimmu.2020.01291)

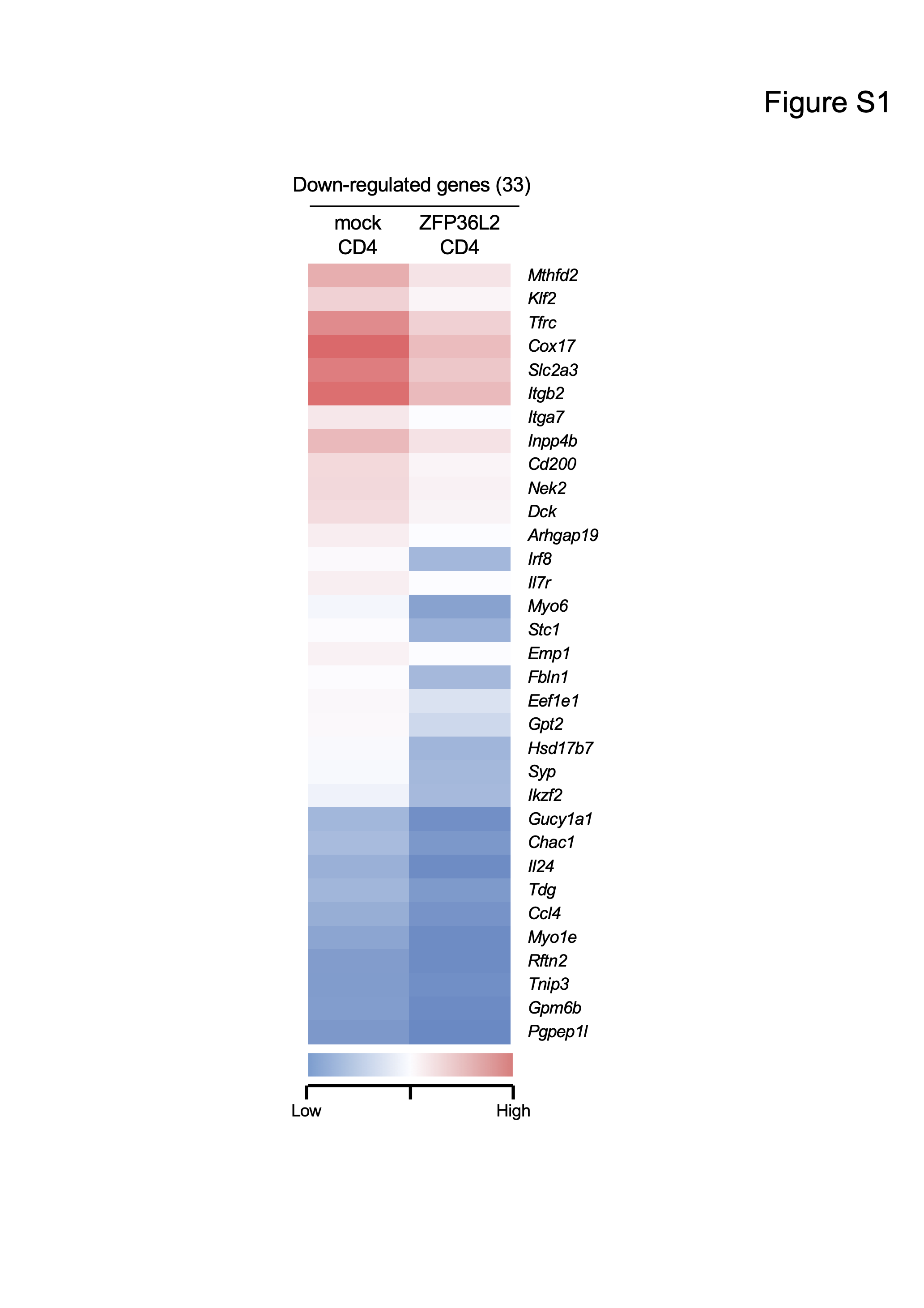

Supplement: Figure S1 — Genes whose expression is downregulated by the forced expression of ZFP36L2. Shown is a heat map of the 33 differentially expressed mRNAs described in Figure 2B. [file Image_1.TIFF]
